# Premedication with intranasal dexmedetomidine decreases barbiturate requirement in pediatric patients sedated for magnetic resonance imaging: a retrospective study

**DOI:** 10.1186/s12871-019-0690-1

**Published:** 2019-02-13

**Authors:** Panu Uusalo, Mirjam Lehtinen, Eliisa Löyttyniemi, Tuula Manner, Mika Scheinin, Teijo I. Saari

**Affiliations:** 1 Department of Anaesthesiology and Intensive Care, University of Turku, P.O. Box 51, Kiinamyllynkatu 4-8, FI-20521 Turku, Finland; 2 Division of Perioperative Services, Intensive Care and Pain Medicine, Turku University Hospital, Turku, Finland; 3 Department of Biostatistics, University of Turku, Turku, Finland; 4 Institute of Biomedicine, University of Turku, Unit of Clinical Pharmacology, Turku University Hospital, Turku, Finland

**Keywords:** MRI, Dexmedetomidine, Thiopental, Pediatric, Sedation

## Abstract

**Background:**

Barbiturates are commonly used in ambulatory sedation of pediatric patients. However, use of barbiturates involve risks of respiratory complications. Dexmedetomidine, a highly selective α_2_-adrenoceptor agonist, is increasingly used for pediatric sedation. Premedication with intranasal (IN) dexmedetomidine offers a non-invasive and efficient possibility to sedate pediatric patients undergoing magnetic resonance imaging (MRI). Our hypothesis was that dexmedetomidine would reduce barbiturate requirements in procedural sedation.

**Methods:**

We included 200 consecutive pediatric patients undergoing MRI, and analyzed their hospital records retrospectively. Half of the patients received 3 μg/kg of IN dexmedetomidine (DEX group) 45–60 min before MRI while the rest received only thiopental (THIO group) for procedural sedation. Sedation was maintained with further intravenous thiopental dosing as needed. Thiopental consumption, heart rate (HR) and peripheral oxygen saturation were recorded.

**Results:**

The cumulative thiopental requirement during MRI was (median and interquartile range [IQR]) 4.4 (2.7–6.0) mg/kg/h in the DEX group and 12.4 (9.8–14.8) mg/kg/h in the THIO group (difference 7.9 mg/kg/h, 95% CI 6.8–8.8, *P* <  0.001). Lowest measured peripheral oxygen saturation remained slightly higher in the DEX group compared to the THIO group (median nadirs and IQR: 97 (95–97) % and 96 (94–97) %, *P* <  0.001). Supplemental oxygen was delivered to 33% of the patients in the THIO group compared to 2% in the DEX group (*P* <  0.001). The lowest measured HR (mean and SD) was lower (78 (16) bpm) in the DEX group compared to the THIO group (92 (12) bpm) (*P* <  0.001).

**Conclusion:**

Premedication with IN dexmedetomidine (3 μg/kg) was associated with markedly reduced thiopental dosage needed for efficient procedural sedation for pediatric MRI.

**Electronic supplementary material:**

The online version of this article (10.1186/s12871-019-0690-1) contains supplementary material, which is available to authorized users.

## Background

Children undergoing magnetic resonance imaging (MRI) are expected to lie immobile in a dimmed, noisy and narrow tunnel, which may cause anxiety and fear. Thus, most of them need procedural sedation. An ideal sedation protocol should have minimal effects on respiration and hemodynamics, maintain the children calm and immobile during the procedure, but allow rapid recovery and discharge.

There are many MRI sedation protocols available for pediatric patients, employing traditional anesthetics such as propofol, barbiturates, benzodiazepines, chloral hydrate, ketamine, remifentanil or sevoflurane [1–3]. Most of these anesthetics may cause respiratory depression or hypotension, which in extreme cases are harmful to the child [2, 4]. Sedation with propofol and remifentanil as well as sedation with sevoflurane may require mechanical ventilation, but also other sedation protocols involve risks of respiratory complications when performed with spontaneous breathing [1, 3].

Dexmedetomidine is an α_2_-adrenoceptor-activating drug used in sedation of adult intensive care patients. In addition to its sedative effects, dexmedetomidine has analgesic and antiemetic effects [5]. Compared to conventional anesthetic agents, patients sedated with dexmedetomidine remain arousable [6]. Dexmedetomidine also has only minimal effects on respiration [7, 8]. Due to its many beneficial properties, dexmedetomidine is currently quite commonly employed in off-label use in pediatric intensive care [9]. In addition, dexmedetomidine has been used in children for other purposes, such as MRI sedation, and several previous reports describe its use in ambulatory sedation of pediatric patients [10–13].

Dexmedetomidine has been shown to reduce the requirements for intravenous anesthetics [14, 15], volatile anesthetics [16, 17] and opioids [18, 19]. In pediatric patients undergoing MRI, premedication with dexmedetomidine reduced propofol consumption, but did not alone cause sufficient sedation in most patients [20, 21]. In adults, dexmedetomidine reduced the dose of thiopental needed for sedation [14, 22], but to our knowledge there are no reports on the effect of dexmedetomidine on the need of barbiturates in sedation of pediatric patients.

Thiopental has been used for decades in sedation of pediatric patients for MRI and recently reported to be a safe and efficacious ambulatory sedative agent for children [1, 2, 23–25]. In Turku University Hospital (TUH) thiopental has been used over 20 years for procedural sedation of pediatric patients undergoing MRI. Sedation with thiopental has mainly been conducted without ventilatory support. In the autumn of 2016, TUH started to use intranasal (IN) dexmedetomidine as premedication for all children scheduled for MRI to reduce the amount of other sedatives. Our primary aim in this study was to compare thiopental requirements after IN dexmedetomidine premedication was added to pediatric MRI sedation protocol. Our secondary aims were to compare the lowest HR and SpO_2_ values recorded during the MRI, and the need for supplemental oxygen during the MRI. We hypothesized that IN dexmedetomidine would markedly reduce the amount of additional sedatives needed for MRI sedation, thus further reducing the risk of respiratory depression.

## Methods

The study protocol was approved by the Hospital District of South-West Finland (T252/2017).

### Patient population

Pediatric patients with normal growth (SD -1.5-1.5), aged between 1 month and 11 years, ASA (American Society of Anesthesiology) status I-II, scheduled for MRI and receiving either thiopental or dexmedetomidine and thiopental for procedural MRI sedation were included in this retrospective analysis. Patients receiving other sedatives than thiopental or dexmedetomidine for MRI and patients with previous exposure to dexmedetomidine within 14 days prior to the index episode, any clinically relevant concomitant drug therapy (e.g. CYP inducers, stimulants), or clinically significant abnormalities in medical examination, ECG or laboratory values were excluded. Eligible patients were identified and patient data were retrieved from the anesthesia reports and patient database of the hospital. One hundred consecutive patients who met the inclusion criteria and received thiopental sedation for MRI were identified between November 2014 and May 2015 (THIO group), and another 100 consecutive patients who had received premedication with IN dexmedetomidine before procedural MRI sedation between January and June 2017 were included in the DEX group. A 50% reduction in thiopental consumption was to be considered clinically significant.

### Diagnostic imaging

MRI was performed using an 1.5 T MRI scanner (Siemens Avanto, Siemens, Erlangen, Germany) according to standard protocols.

### Drug administration

The dose of IN dexmedetomidine (dexmedetomidine hydrochloride 100 μg/ml, Dexdor®, Orion Pharma, Finland) used in the DEX group (3 μg/kg) was based on previous reports [10, 11] attesting to the safety of intravenous dexmedetomidine at doses up to 9 μg/kg given over 30 min. The individual dose was rounded to the nearest ten micrograms to facilitate dosing. IN dexmedetomidine was administered with an LMA MAD Nasal™ device (Teleflex MAD Nasal: Research Triangle Park, NC, USA) approximately 45 to 60 min prior to the planned MRI procedure. No other sedative medication was used. The patients of the THIO group received no sedative premedication. Their treatment was in other respects identical to that of the patients of the DEX group.

Before transfer to the MRI room, a venous cannula (B. Braun, Melsungen, Germany) was inserted in a suitable forearm vein. EMLA cream (lidocaine 2.5% and prilocaine 2.5%, AstraZeneca Inc., Södertälje, Sweden) was applied to some patients to facilitate the venous cannulation.

In the MRI room, the patients received intravenous thiopental (thiopental sodium 25 mg·ml^− 1^, Pentocur®, Abcur, Helsingborg, Sweden) for sedation, as required. The decision to administer thiopental as well as the selection of the individual dose of thiopental was determined by the anesthetist in charge of the patient during the MRI procedure, and was based on the MOAA/S (Modified Observer’s Assesment of Alertness/Sedation Scale). The aim of sedation was to keep the MOAA/S (Modified Observer’s Assesment of Alertness/Sedation Scale) -level of the patients between 1 and 3.

### Pharmacodynamic measurements

Vital signs were monitored continuously with pulse oximetry (SpO_2_) and electrocardiography after dexmedetomidine administration in the DEX group, and from administration of thiopental until at least one hour after MRI in both groups.

### Oxygen administration

The decision to deliver supplemental oxygen was based on SpO2 levels lower than 94%. Oxygen was delivered with the “blow by” method, which is commonly used in sedated spontaneously breathing children [26], with a flow of 4 to 6 l/min.

### Time to discharge

Patients fulfilled the discharge criteria when they were able to drink and eat. For safety and ethical reasons all patients were observed at least 2 h after the end of MRI. Time to discharge from the MRI unit was defined as the period of time between the end of the MRI procedure and the time of discharge. Clock times were obtained from the hospital’s patient information system.

### Adverse events

Patient data were collected from the hospital’s patient information system and anesthesia reports, and possible adverse events (e.g. nausea and vomiting) related to the procedures were manually identified.

### Statistics

The sample size was based on previous experience in similar studies [20, 21]. The primary outcome variable was the amount of thiopental administered to the patients (induction dose (mg/kg) and consumption of thiopental per hour (mg/kg/h). Secondary outcomes were the lowest HR values recorded during the MRI, lowest SpO_2_ values during the MRI, the need of supplemental oxygen during the MRI and adverse events. The Shapiro-Wilk test (*P* > 0.05) was used to assess normality assumptions. Student’s t-test was used to compare the groups with normally distributed data, and Wilcoxon’s rank sum test was used to test non-normally distributed data. Primary outcomes were tested using the Kruskal-Wallis test and continued with age group comparisons (corrected with the Steel-Dwass method). Nominal data were tested using chi-square analysis. *P* <  0.05 (two-tailed) was considered statistically significant. The results are expressed as mean values with standard deviations (SD), and as medians with interquartile ranges (IQR) when the normality assumption was not met. The analyses were performed with JMP Pro 13.0 and SAS® System programs, version 9.4 for Windows (SAS Institute Inc., Cary, NC, USA).

## Results

One hundred consecutive patients were included in both study groups (DEX and THIO) (Additional file 1: Figure S1). These groups were subdivided into three clinically relevant age groups for statistical analysis, THIO1 and DEX1 (0–2 years), THIO2 and DEX2 (2–6 years) and THIO3 and DEX3 (6–11 years). Patient descriptors are shown in Table 1. The DEX patients had a mean (SD) age of 4.50 (2.56) years and a mean body-mass index (BMI) of 16.5 (2.2) kg/m^2^, and the THIO patients had a mean (SD) age of 4.06 (2.39) years and a mean BMI of 16.5 (1.8) kg/m^2^. The median (range) dose of dexmedetomidine was 50 (20–110) ug.

**Table 1:** Patient characteristics

	Group	Dexmedetomidine (DEX)		Thiopental (THIO)		*p*-value
		n	mean (SD)^a^	n	mean (SD)^a^	
Age (yr)	ALL	100	4.50 (2.56)	100	4.06 (2.39)	0.22
	0–2 years	20	1.21 (0.56)	24	1.24 (0.45)	
	2–6 years	50	3.93 (1.16)	53	3.85 (1.09)	
	6–11 years	30	7.64 (1.30)	23	7.47 (1.31)	
Weight (kg)	ALL	100	18.8 (7.5)	100	17.4 (6.4)	0.19
	0–2 years	20	10.3 (1.9)	24	10.5 (2.1)	
	2–6 years	50	17.4 (3.3)	53	17.0 (3.4)	
	6–11 years	30	26.8 (7.1)	23	25.6 (5.7)	
BMI (kg/m^2^)	ALL	100	16.5 (2.2)	100	16.5 (1.8)	0.68
	0–2 years	20	17.4 (2.1)	24	17.3 (2.2)	
	2–6 years	50	16.3 (2.0)	53	16.5 (1.6)	
	6–11 years	30	16.3 (2.4)	23	15.9 (1.7)	
Duration of MRI (min)	ALL	100	43 (41–57)	100	47 (35–50)	0.001
	0–2 years	20	43 (36–57)	24	49 (43–60)	0.124
	2–6 years	50	42 (35–48)	53	46 (40–57)	0.002
	6–11 years	30	43 (34–55)	23	50 (37–55)	0.804

^a^Mean and SD reported, except for duration of MRI, for which median and IQR are shown

The MRI duration was somewhat shorter in the DEX group compared to the THIO group (medians 43 and 47 min, IQR 41–57 and 35–50 min; *P* <  0.001). There were no other statistically significant differences in the patient characteristics between the two groups. Diagnostic categories and MRI types are shown in a (Additional file 2: Table S1).

**Fig. 1:**
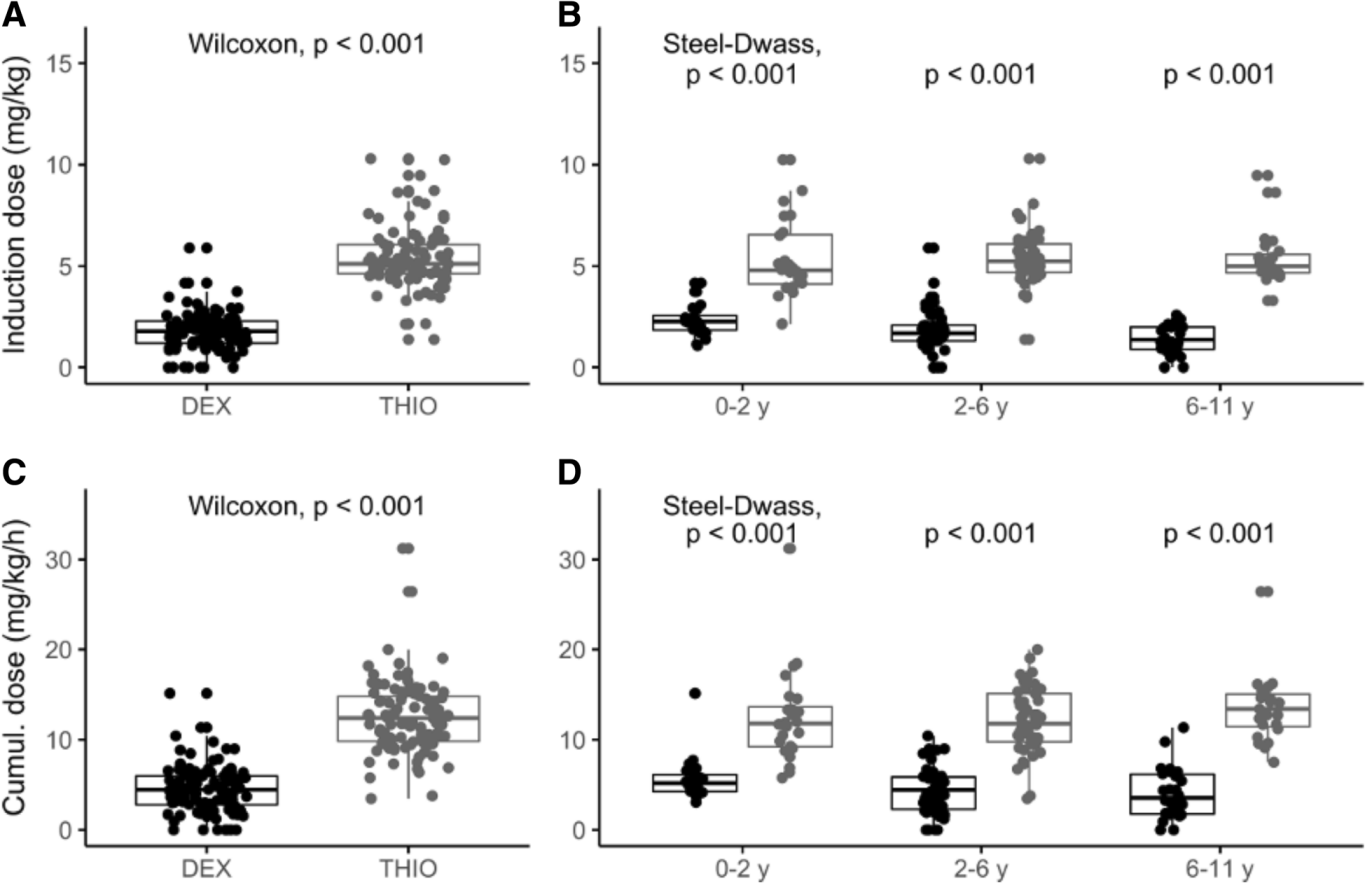
Induction doses and cumulative dosing of thiopental in three clinically relevant age groups. Difference between dexmedetomidine (DEX) and thiopental (TIO) groups was tested with Wilcoxon test for induction dose of thiopental (mg/kg) (**a**) and for cumulative dose of thiopental (mg/kg/h) (**c**). Difference in induction dose of thiopental (mg/kg) (**b**) and cumulative dose of thiopental (mg/kg/h) (**d**) between three clinically significant age groups of dexmedetomidine group (DEX1, DEX2 and DEX3) and thiopental group (THIO1, THIO2, THIO3) were tested using Kruskal-Wallis test and continued with pairwise comparisons which were corrected with Steel-Dwass method for all pairs

Significantly smaller induction and total doses of thiopental were needed for completion of MRI in all DEX age groups compared to the THIO age groups. The median (IQR) cumulative thiopental requirement during MRI was 4.4 (2.7–6.0) mg/kg/h compared to 12.4 (9.8–14.8) mg/kg/h in DEX and THIO groups, respectively (median difference 7.9 mg/kg/h, 95% CI: 6.8–8.8, *P* <  0.001). The median (IQR) induction doses of thiopental before MRI were 1.8 (1.2–2.3) mg/kg compared to 5.1 (4.6–6.2) mg/kg in the DEX and THIO groups, respectively (median difference 3.3 mg/kg/h, 95% CI: 3.1–3.6, *P* <  0.001). Differences in thiopental requirements were statistically significant between all three DEX and THIO age groups (*P* <  0.001) (Fig. 1 and Table 2).

**Table 2:** Dosing of thiopental for procedural MRI sedation

	Group	Dexmedetomidine (DEX)		Thiopental (THIO)		
		n	median (IQR)	n	median (IQR)	*p*-value
Thiopental induction dose (mg/kg)	ALL	100	1.8 (1.2–2.3)	100	5.1 (4.6–6.1)	< 0.001
	0–2 years	20	2.3 (1.8–2.8)	24	4.8 (4.0–6.6)	< 0.001
	2–6 years	50	1.7 (1.3–2.2)	53	5.2 (4.7–6.1)	< 0.001
	6–11 years	30	1.4 (0.9–2.0)	23	5.0 (4.7–5.7)	< 0.001
Thiopental cumulative dose (mg/kg/h)	ALL	100	4.4 (2.7–6.0)	100	12.4 (9.8–14.8)	< 0.001
	0–2 years	20	5.2 (4.2–6.4)	24	11.8 (9.1–14.2)	< 0.001
	2–6 years	50	4.4 (2.2–5.9)	53	11.8 (9.7–15.2)	< 0.001
	6–11 years	30	3.5 (1.7–6.3)	23	13.4 (11.2–15.3)	< 0.001

The median (IQR) of the lowest observed peripheral oxygen saturation reading was 97 (95–97) % in the DEX group compared to 96 (94–97) % in the THIO group (*P* <  0.001). This analysis did not take into account the use of supplemental oxygen, but the reported values are after possible oxygen administration. Thirty-three (33%) of the patients in the THIO group received supplemental “blow by” oxygen whereas only two (2%) the DEX patients received oxygen (*P* <  0.001). The median (IQR) time from the end of MRI to discharge was 140 (115–169) min in the DEX group compared with 150 (125–175) min in the THIO group (*P* = 0.15). The mean (SD) of the lowest measured HR was 78 (16) bpm in the DEX group compared to 92 (12) bpm in the THIO group (*P* = 0.0001) (Table 3).

**Table 3:** Vital signs during MRI, discharge times and use of supplemental oxygen

	Group	Dexmedetomidine (DEX)		Thiopental (THIO)		*p*-value
		n	median (IQR)	n	median (IQR)	
Lowest SpO_2_ (%) during MRI	ALL	100	97 (95–97)	100	96 (94–97)	< 0.001
	0–2 years	20	96 (95–97)	24	96 (94–97)	0.863
	2–6 years	50	97 (96–98)	53	95 (94–97)	0.002
	6–11 years	30	97 (96–98)	23	96 (94–97)	0.114
Lowest heart rate (1/min) during MRI	ALL	100	78 (16)	100	92 (12)	< 0.001
	0–2 years	20	97 (11)	24	104 (13)	0.056
	2–6 years	50	77 (12)	53	91 (9)	< 0.001
	6–11 years	30	65 (12)	23	82 (8)	< 0.001
Time from MRI end to discharge (min)	ALL	100	140 (115–169)	100	150 (125–175)	0.15
	0–2 years	20	151 (125–184)	24	144 (123–162)	
	2–6 years	50	142 (122–172)	53	152 (131–177)	
	6–11 years	30	124 (104–146)	23	139 (114–172)	
			n (%)		n (%)	
Supplemental oxygen (%)	ALL	100	2 (2)	100	33 (33)	< 0.001
	0–2 years	20	2 (10)	24	9 (38)	0.04
	2–6 years	50	0 (0)	53	19 (36)	< 0.001
	6–11 years	30	0 (0)	23	5 (22)	0.003

Median and IQR reported, except for lowest heart rate, for which mean and SD is shown. Number and percentage of patients receiving supplemental oxygen is shown

### Adverse events

Two patients (aged 4.7 and 7.8 years) in the DEX group received atropine for bradycardia (36 and 39 /min respectively) after thiopental administration. Two patients in the DEX group and two patients in the THIO group reported mild nausea after MRI. No other adverse events were recorded.

## Discussion

To our knowledge, this is the first study to investigate the impact of premedication with IN dexmedetomidine on barbiturate requirements for procedural MRI sedation of pediatric patients. We compared two different pediatric MRI sedation protocols, employing retrospective analysis of hospital records. Our hypothesis was that the requirement of intravenous thiopental would decrease when IN dexmedetomidine is used as premedication. Our results indicate that significantly less thiopental is needed for pediatric procedural MRI sedation when IN dexmedetomidine is used as premedication.

We hypothesized that use of dexmedetomidine as premedication could reduce the risk of respiratory depression caused by thiopental, which was seen as reduced need for supplemental oxygen. Despite 33% of patients in THIO-group and only 2% of patients in DEX-group received supplemental oxygen, lower peripheral oxygen saturations were measured in THIO-group. It seems that patients receiving dexmedetomidine as premedication do not mostly need supplemental oxygen, which may protect patients from hypercapnia and respiratory depression that oxygen administration may cause.

It is well known that dexmedetomidine decreases heart rate due its sympatholytic effects [8, 10–12]. In our study two patients in DEX-group received atropine for bradycardia, which, however, only emerged first after thiopental administration. It has been suggested in the literature that liberal correction of bradycardia induced by alpha-2-agonists may cause hypertensive crisis among pediatric patients [27, 28]. However patients sedated with dexmedetomidine and furthermore receiving other sedatives must be carefully monitored for adverse events such as bradycardia. Blood pressure was not measured routinely in the patients included in our study, and was thus not included in the analysis. However, the effect of dexmedetomidine on mean arterial blood pressure levels of pediatric patients has been widely studied and it has been shown that intranasal dosages of 3 μg/kg do not cause clinically significant hypotension or hypoperfusion [13]. Instead, hypertension may result, especially when higher dosages of dexmedetomidine are used in pediatric patients sedated for MRI. Still, a previous study with over 3500 pediatric patients showed that the incidence of hypertension is low [29].

The duration of MRI was shorter in DEX group compared to THIO-group. Head MRI was the most common MRI type (*n* = 52 and *n* = 59) in DEX and THIO group, respectively. The mean time of head MRI was shorter in DEX group (42 vs 47 min). Despite all imaging were performed with same Siemens 1.5 T MRI scanner, there has been an software update of the scanner in year 2017, which may have reduced the length of head MRI. Indications for MRI scan varied in our patient group which partly explain the variability in the time of MRI. Furthermore in some occasions the sedation was not sufficient and some scans were repeated after additional doses of thiopental were administered to complete the MRI. Thus we cannot draw any conclusions that use of dexmedetomidine as premedication would reduce the length of MRI. There was no statistically significant difference in time to discharge between two groups. For safety and ethical reasons all patients were observed at least 2 h after the end of MRI, despite all patients were alert soon after the end of MRI.

Sedation with dexmedetomidine resembles physiological sleep and maintains the patients arousable. This may be challenging in procedures where the patients are expected to remain immobile. On the other hand, patients should be arousable soon after MRI to be discharged from the imaging unit. Almost all (98 of 100) of our DEX patients received thiopental due to emergence during transfer to the MRI room. To minimize the need of additional sedation at this time point, it would be worthwhile to wait until the patients again calm down on the MRI bed. After our clinic started to employ IN dexmedetomidine as a routine premedication for pediatric patients undergoing MRI, the need for thiopental appears to have markedly decreased. Considering the respiratory depression, nausea and other adverse effects that barbiturates may cause, the reduced consumption of thiopental is probably favorable and may reduce the risk of adverse events in sedation of pediatric patients for MRI. However, the use of IN dexmedetomidine as premedication appears not to totally remove the further need for gamma aminobutyric acid agonists.

To our knowledge there are two reports on the impact of dexmedetomidine premedication on the propofol consumption in pediatric patients undergoing MRI. In a retrospective study of Boriosi et al. (2017) 256 ASA-class 1–2 pediatric patients were sedated for MRI with propofol and two third of them received 1–2 μg/kg of intravenous dexmedetomidine before MRI. Both groups received oral midazolam (0.3–0.5 mg/kg) as premedication. There was reduced propofol consumption (*P* <  0.001) and less adverse events (*P* = 0.008) in dexmedetomidine group. 12% of patients needed interventions on airway and 4% of patients had upper airway obstruction in dexmedetomidine group [20]. In the study of Gyanesh et al. (2014), the use of IN dexmedetomidine (1 μg/kg) as premedication (*n* = 52) reduced propofol dose (*P* <  0.001), duration of awakening (*P* <  0.001) and duration of discharge (*P* <  0.001) compared to the use of IN saline as premedication (*n* = 46) in children aged 1 to 10 years undergoing MRI. All patients were breathing spontaneously and no artificial airway was needed [21].

Use of IN dexmedetomidine in combination with midazolam has also been shown to be effective way to maintain patient satisfaction, adequate respiration and stable hemodynamics in MRI sedation of pediatric patients [30]. In the era of multimodal anesthesia it appears that the use of two sedative agents can provide a safer way for sedating pediatric patients undergoing MRI with respect to adequate respiration and hemodynamic control [1].

Thiopental has been used in our unit successfully for decades in sedation of pediatric patients undergoing MRI. Children, on the average, eliminate thiopental more rapidly than adults, but the elimination half-life of thiopental is still quite long, from 6 to 12 h [31, 32]. Artificial airway and mechanical ventilation have been warranted only in rare cases (e.g. aspiration risk or suspected impaired consciousness) and dosing of 10–15 mg/kg/h of intravenous thiopental has allowed the patients to maintain spontaneous, unassisted respiration and rapid recovery after the MRI. However, supplemental oxygen has been administered frequently to the patients.

Our study has obvious limitations. A relatively new sedation protocol was used when the patients in the DEX group were treated. This might have influenced the outcome as the personnel sedating children for MRI were still collecting experience on the use of IN dexmedetomidine. On the other hand, the same few pediatric anesthesiologists were in charge of all of the patients. Another limitation is the retrospective design of this study, which could have affected the results, even when consecutive patients were collected in order to avoid any selection bias.

## Conclusion

IN dexmedetomidine is an easily administered premedication agent or anesthetic adjuvant that appears to markedly reduce the requirement of barbiturates. Compared to sedation with thiopental alone, the use of IN dexmedetomidine as premedication may help to prevent respiratory depression. Further studies on the optimal dosage and delivery methods of IN dexmedetomidine for procedural MRI sedation appear as warranted.

## Additional file

Additional file 1: **Figure S1.** Flow diagram of the study. (PDF 172 kb)

Additional file 2: **Table 1**. Diagnostic categories and the type of MRI study conducted for the patients. Patients in DEX-group received intranasal administration of 3 μg/kg dexmedetomidine before magnetic resonance imaging, while patients in THIO group received no premedication before MRI. (PDF 24 kb)

## Abbreviations

DEX: Dexmedetomidine group
IN: Intranasal
MRI: Magnetic resonance imaging
SpO2: Pulse oximetry
THIO: Thiopental group
TUH: Turku University Hospital
