# Supplementary figures and images for: Premedication with intranasal dexmedetomidine decreases barbiturate requirement in pediatric patients sedated for magnetic resonance imaging: a retrospective study
Source: BMC Anesthesiol. 2019 Feb 13;19:22. doi: 10.1186/s12871-019-0690-1 (PMC6374898; doi:10.1186/s12871-019-0690-1)

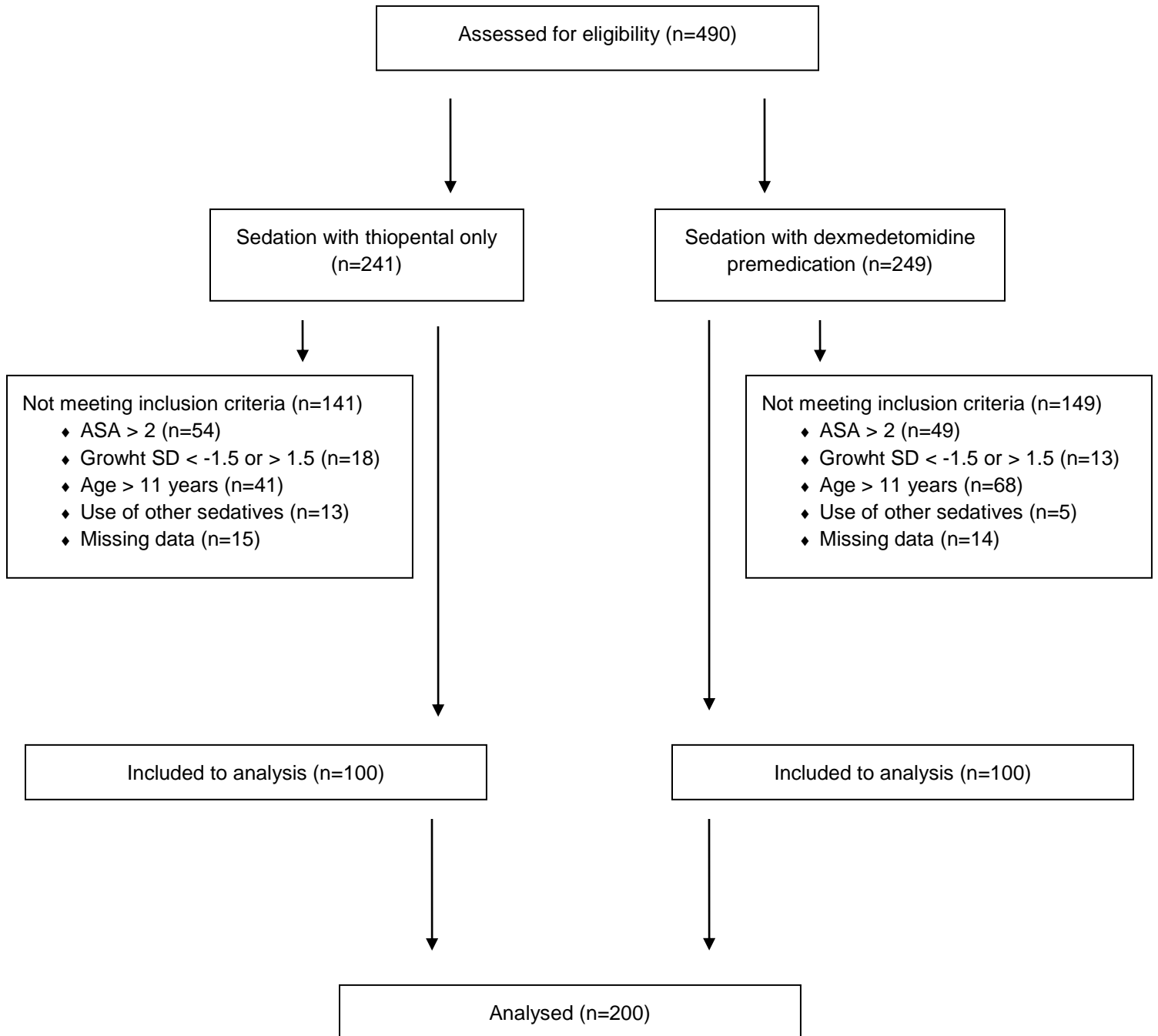

Supplement: Supplementary file 1 — Figure S1. Flow diagram of the study. (PDF 172 kb) [file 12871_2019_690_MOESM1_ESM.pdf]
