# Supplementary material for: Premedication with intranasal dexmedetomidine decreases barbiturate requirement in pediatric patients sedated for magnetic resonance imaging: a retrospective study
Source: BMC Anesthesiol. 2019 Feb 13;19:22. doi: 10.1186/s12871-019-0690-1 (PMC6374898; doi:10.1186/s12871-019-0690-1)
Supplement: Supplementary file 2 — Table 1. Diagnostic categories and the type of MRI study conducted for the patients. Patients in DEX-group received intranasal administration of 3 μg/kg dexmedetomidine before magnetic resonance imaging, while patients in THIO group received no premedication before MRI. (PDF 24 kb) [file 12871_2019_690_MOESM2_ESM.pdf]

Supplemental Table 1: Diagnostic categories and the type of MRI study conducted for the patients. Patients in DEX-group received intranasal administration of 3 µg/kg dexmedetomidine before magnetic resonance imaging, while patients in THIO group received no premedication before MRI.

	Dexmedetomidine				Thiopental			
	DEX ALL	DEX1	DEX2	DEX3	THIO ALL	THIO1	THIO2	THIO3
**Diagnostic categories (n)**	100	20	50	30	100	24	53	23
Neurology	21	3	11	7	24	5	11	8
Neurosurgery	7	2	4	1	8	1	7	0
Ophthalmology	4	1	3	0	4	0	3	1
Hematology/Oncology	8	0	3	5	7	1	*5*	1
Genetic	5	0	2	3	8	2	5	1
Rheumatologic	17	3	9	5	12	0	5	7
Trauma	16	3	9	4	11	6	4	1
Other	22	8	9	5	26	9	13	4
**Type of MRI (n)**								
Head	52	11	26	15	59	16	30	13
Extremity	21	4	11	6	16	3	8	5
Spine	8	2	4	2	7	1	3	3
Pelvis	5	1	3	1	4	1	2	1
Whole body	3	0	1	2	7	2	4	1
Abdomen	7	2	2	3	3	1	2	0
Other	4	0	3	1	4	0	4	0
